# Bridging clinical informatics and implementation science to improve cancer symptom management in ambulatory oncology practices: experiences from the IMPACT consortium

**DOI:** 10.1093/jamiaopen/ooae081

**Published:** 2024-09-04

**Authors:** Nadine Jackson McCleary, James L Merle, Joshua E Richardson, Michael Bass, Sofia F Garcia, Andrea L Cheville, Sandra A Mitchell, Roxanne Jensen, Sarah Minteer, Jessica D Austin, Nathan Tesch, Lisa DiMartino, Michael J Hassett, Raymond U Osarogiagbon, Sandra Wong, Deborah Schrag, David Cella, Ashley Wilder Smith, Justin D Smith, IMPACT Consortium, David Cella, Andrea Cheville, Michael J Hassett, Raymond U Osarogiagbon, Deborah Schrag, Sandra L Wong, Barbara L Kroner, Ashley Wilder Smith, Lisa DiMartino, Sofia Garcia, Joan Griffin, Roxanne Jensen, Sandra Mitchell, Kathryn Ruddy, Justin D Smith, Betina Yanez, Jessica J Bian, Don S Dizon, Hannah W Hazard-Jenkins, Mary-Anne Ardini, Paige Ahrens, Jessica Austin, Fiona Barrett, Michael Bass, Megan Begnoche, September Cahue, Kimberly Caron, Linda Chlan, Ava Coughlin, Christine Cronin, Samira Dias, Nicolas Faris, Anne Marie Flores, Martha Garcia, Karla Hemming, Jeph Herrin, Christine Hodgdon, Sheetal Kircher, Kurt Kroenke, Veronica Lam, Nicola Lancki, Quan H Mai, Jennifer Mallow, Nadine J McCleary, Wynne Norton, Mary O'Connor, Deirdre Pachman, Loretta Pearson, Frank Penedo, Jewel Podratz, Jennifer Popovic, Liliana Preiss, Parvez Rahman, Sarah Redmond, James Reich, Joshua Richardson, Kimberly Richardson, Jennifer Ridgeway, Lila Rutten, Karen Schaepe, Denise Scholtens, Tiana Poirier-Shelton, Philip Silberman, Jaclyn Simpson, Laura Tasker, Nathan Tesch, Cindy Tofthagen, Angela Tramontano, Benjamin D Tyndall, Hajime Uno, Firas Wehbe, Bryan Weiner

**Affiliations:** Department of Medical Oncology and Division of Population Sciences, Dana-Farber Cancer Institute, Boston, MA 02115, United States; Division of Health System Innovation and Research, Department of Population Health Sciences, Spencer Fox Eccles School of Medicine, University of Utah, Salt Lake City, UT 84132, United States; Galter Health Sciences Library and Learning Center, Northwestern University Feinberg School of Medicine, Chicago, IL 60611, United States; Department of Medical Social Science, Northwestern University, Chicago, IL 60611, United States; Department of Medical Social Science, Northwestern University, Chicago, IL 60611, United States; Department of Physical Medicine & Rehabilitation, Mayo Clinic, MN 55905, United States; Outcomes Research Branch, Division of Cancer Control and Population Sciences, National Cancer Institute, Rockville, MD 20850, United States; Outcomes Research Branch, Division of Cancer Control and Population Sciences, National Cancer Institute, Rockville, MD 20850, United States; Department of Physical Medicine & Rehabilitation, Mayo Clinic, MN 55905, United States; Division of Epidemiology, Department of Quantitative Health Sciences, Mayo Clinic Arizona, Mayo Clinic Cancer Center, Population Sciences Program, Scottsdale, AZ 85054, United States; Robert D. and Patricia E. Kern Center for the Science of Healthcare Delivery, Mayo Clinic, MN 55905, United States; University of Texas Southwestern Medical Center, Dallas, TX 75390, United States; RTI International, Research Triangle Park, NC 27709, United States; Department of Medical Oncology and Division of Population Sciences, Dana-Farber Cancer Institute, Boston, MA 02115, United States; Baptist Medical Center, Memphis, TN 38120, United States; Department of Surgery, Dartmouth Hitchcock Medical Center, Lebanon, NH 03766, United States; Department of Medicine, Memorial Sloan Kettering Cancer Center, New York, NY 10065, United States; Institute for Public Health and Medicine, Center for Patient-Centered Outcomes, Northwestern University Feinberg School of Medicine, Chicago, IL 60611, United States; Outcomes Research Branch, Division of Cancer Control and Population Sciences, National Cancer Institute, Rockville, MD 20850, United States; Division of Health System Innovation and Research, Department of Population Health Sciences, Spencer Fox Eccles School of Medicine, University of Utah, Salt Lake City, UT 84132, United States; Department of Physical Medicine & Rehabilitation, Mayo Clinic, MN 55905, United States

**Keywords:** implementation science, medical information, symptom management, cancer, patient-reported outcomes, electronic health information

## Abstract

**Objectives:**

To report lessons from integrating the methods and perspectives of clinical informatics (CI) and implementation science (IS) in the context of Improving the Management of symPtoms during and following Cancer Treatment (IMPACT) Consortium pragmatic trials.

**Materials and Methods:**

IMPACT informaticists, trialists, and implementation scientists met to identify challenges and solutions by examining robust case examples from 3 Research Centers that are deploying systematic symptom assessment and management interventions via electronic health records (EHRs). Investigators discussed data collection and CI challenges, implementation strategies, and lessons learned.

**Results:**

CI implementation strategies and EHRs systems were utilized to collect and act upon symptoms and impairments in functioning via electronic patient-reported outcomes (ePRO) captured in ambulatory oncology settings. Limited EHR functionality and data collection capabilities constrained the ability to address IS questions. Collecting ePRO data required significant planning and organizational champions adept at navigating ambiguity.

**Discussion:**

Bringing together CI and IS perspectives offers critical opportunities for monitoring and managing cancer symptoms via ePROs. Discussions between CI and IS researchers identified and addressed gaps between applied informatics implementation and theory-based IS trial and evaluation methods. The use of common terminology may foster shared mental models between CI and IS communities to enhance EHR design to more effectively facilitate ePRO implementation and clinical responses.

**Conclusion:**

Implementation of ePROs in ambulatory oncology clinics benefits from common understanding of the concepts, lexicon, and incentives between CI implementers and IS researchers to facilitate and measure the results of implementation efforts.

## Background and significance

Clinical informatics (CI) is a field that uses “biomedical data, information, and knowledge for scientific inquiry, problem solving, and decision making, motivated by efforts to improve human health.”^1^ However, CI efforts are challenged with system adoption, acceptability, efficiency, and unintended consequences associated with implementation.^2–4^ In addressing those challenges, implementation science (IS) can play a pivotal role in the uptake and sustainment of evidence-based interventions (EBIs) in healthcare, particularly when those EBIs are integrated into electronic health records (EHRs).

CI provides approaches for the development of efficient and effective strategies and tools to support implementation of various EBIs.^5^ CI practitioners often use the “Five Rights Framework” to assess the implementation of clinical decision support (CDS) tools by how it delivers the right information, to the right people, at the right time, in the right format, and within the right workflow.^6^ The Five Rights Framework has served to focus efforts on real-world implementations of CDS and clinical information systems. It has informed evaluations that focus on clinical and economic outcomes and has been a driver for designing rigorous research studies. Similarly, the GUIDES checklist is grounded in informatics and lays out considerations in the development and design of CDS that ultimately affect implementation success.^7^

Increasingly, clinical informaticists have incorporated IS frameworks to inform the development and evaluate the deployment of informatics applications (eg, tools, systems).^8^^,^^9^ For example, Bakken and Ruland found utility in the RE-AIM (Reach, Effectiveness, Adoption, Implementation, Maintenance) framework for evaluating real-world case studies of CDS implementations.^10^ Moreover, CI practitioners and researchers are applying IS methods to guide efforts from learning health systems,^11–13^ to data quality,^14^ to cancer informatics,^15^ the focus of this article.

### Implementation science

IS can help implementers better anticipate, mitigate, and overcome challenges to adoption and sustained use of CI tools.^16^ The use of IS methods can aid in demonstrating the value and impact of CI for healthcare systems and patient outcomes, and with empirical testing, would provide an evidence base. Implementation strategies—the actions, methods, or techniques that enhance implementation of EBIs^17–19^—target implementers and delivery systems to support adoption, effective integration, and sustainment by overcoming known and anticipated implementation barriers. CI tools such as EHR features, digitized clinical practice guidelines (CPGs), and CDS can be considered implementation strategies as they support implementers in the delivery of EBIs. Implementation outcomes (eg, adoption, reach, cost, fidelity, and sustainment^20^) are the effects of strategies and serve as indicators of implementation success.^17^ Implementation outcomes differ from service system (eg, quality of care, equity) or clinical effectiveness outcomes (eg, patient health outcomes), in that they relate to how well and to what extent the EBI was implemented. This is a precondition to determining whether the EBI effectively achieved the desired changes in clinical or service outcomes.^17^

A distinction needs to be made between key related implementation terms and concepts when considering applications or integration with CI approaches. The National Institutes of Health defines implementation *science* as “the study of methods to promote the integration of research findings and evidence into healthcare policy and practice”; implementation *research* “evaluates of the use of strategies to integrate interventions into real-world settings to improve patient outcomes”; and implementation *practice* is “the use of strategies to adopt and integrate evidence-based health interventions and change practice patterns within and across specific systems.”^21^ Implementation research and practice share some overlap with *quality improvement* but have distinct aims and methods.^22^^,^^23^ Implementation research has traditionally focused on testing strategies for establishing evidence-based practices in routine care while quality improvement aims to devise and test solutions for local challenges generally, without the intent or methods needed to create generalizable knowledge. Finally, *implementation practice* is simply the use of implementation strategies in practice settings. Notifying and providing brief training to clinicians when a new tool is enabled in the EHR to encourage use (without a formal test or evaluation of effect) is an example of implementation practice in CI.

### Bridging clinical informatics and implementation science

The fields of CI and IS are both focused on strategies for promoting effective implementation of EBIs; yet each brings its own set of terminologies, methods, practices, and incentives to bear. For example, the word “implementation” itself may be construed differently across CI and IS audiences, where the former may consider aspects of implementing value sets in a CDS tool and an IS audience may consider the multilevel factors that impact whether implementing a CDS tool within a specific clinic setting will be successful. We posit that IS offers clinical informaticists (1) increasingly powerful models and frameworks with which to plan and execute implementations, (2) strategies to promote the sustainable deployment of EBIs and CI tools for wide-scale adoption by healthcare systems, and (3) methods for testing and measuring the effect of these strategies on EBI adoption, reach to eligible patients, and sustainment.

Integration of IS methods could address one of the primary challenges in CI: In a systematic review, Jones et al.^24^ reported that 61% of 236 studies failed to provide adequate implementation context details, making it “impossible to determine why some HIT implementations are successful and others are not.” The report concluded that, “the most important improvement that can be made in HIT evaluations is increased reporting of the effects of implementation and context.” In short, CI tools cannot be effective without meaningful use and better attention to context is key. One of the most commonly used frameworks in IS for describing context is the Consolidated Framework for Implementation Research (CFIR).^25^ This framework specifies contextual determinants of implementation (barriers and facilitators) in 5 domains: Inner Setting, Intervention Characteristic, Individual Characteristic, Process, and Outer Setting. Using a framework like CFIR importantly incorporates the perspectives of key stakeholders which are frequently overlooked in CI implementations; namely: patients, clinicians, staff, administrators, funders, etc. In the literature, CFIR has been used to identify and address multilevel contextual determinants specifically for ePROs in healthcare settings.^26^

Our interventions have encountered these challenges during the conduct of the ***I****mproving the****M****anagement of sym****P****toms during****A****nd following****C****ancer****T****reatment* (*IMPACT*) Consortium funded by the National Cancer Institute (NCI) under the Cancer Moonshot^SM^.^27^ IMPACT was designed to support the development, implementation, evaluation, and scaling of EHR-integrated electronic symptom management systems, using IS methods (ie, models/frameworks, study designs, evaluation, implementation strategies). IMPACT deploys interventions that (1) systematically monitor patient-reported symptoms; (2) trigger clinician responses consistent with evidence-based guidelines; and (3) provide support options such as CDS. Approaches are tested via pragmatic randomized trials.

## Objective

We describe lessons learned from integrating the objectives and methods of CI and IS to achieve improved monitoring and management of cancer patient symptoms through EHR-delivered interventions. The goal of this manuscript is to describe the use of informatics-based tools as implementation strategies to support delivery and sustainment of these interventions to improve cancer outcomes. Insights at the intersection of CI and IS are appraised to support future research and implementation efforts.

## Materials and methods

IMPACT is comprised of 3 large, diverse Research Centers (RCs) conducting hybrid effectiveness-implementation studies using randomized trial designs.^28^ The RCs are (1) Northwestern University IMPACT (NU IMPACT) in Chicago, IL; Enhanced Electronic Health Record Facilitated Cancer Symptom Control (E2C2) in Rochester, MN^29^; and Symptom Management Implementation of Patient Reported Outcomes in Oncology (SIMPRO) in 6 health systems in 6 states.^30^ Each RC is implementing a cancer symptom surveillance (involving ePROs) and evidence-based symptom management intervention in ambulatory oncology practices. Numerous CI tools embedded in EHRs are being used as implementation strategies, including prompts/alerts to the care team, dashboards with graphic displays of ePRO data, automated prompts to patients for ePRO completion, alerts to patients to contact care team when severe symptoms are reported, CDS, and audit and feedback systems. IMPACT RCs are using pragmatic research designs and rigorous scientific methods to ensure generalizability of the implementation strategies for systems adopting these interventions in the future.

We posit that clinical informatics and implementation science each offer a distinct yet complementary set of theories and methods to support an evidence-based approach to healthcare delivery and the adoption of health-related information and communication technologies.^31–33^ In this paper, we refer to clinical informatics as an applied field integrating information technology and data science that is designed to (i) capture data streams at the point of care; (ii) aggregate, synthesize, and present those data to clinicians and decision-makers in a manner that facilitates actionability, a high reliability care delivery system, and improved clinical outcomes; (iii) provide tools that make decision-making more efficient, timely, and evidence-informed; and (iv) make available large datasets for the generation of new knowledge.

Implementation science is a research framework that systematically (i) addresses the barriers that prevent or slow the uptake of effective research-tested interventions into regular use by practitioners and policymakers; (ii) improves the reach, acceptability, and patient/community engagement that is necessary for access and adoption; and (iii) ensures that with sustained implementation, including adaption to variations in care delivery contexts, clinician workflows, organizational resources, and patient preferences, the effectiveness of an evidence-based intervention is not eroded.

Both clinical informatics and implementation science utilize cyclical action research processes,^34^ involve stakeholders as active collaborators, and have as their overall goal the improvement of patient experiences of care and population health, conservation of costs, and promotion of continuous learning.^34–36^ In the IMPACT consortium, we conducted 3 distinct pragmatic trials integrating clinical informatics and implementation science. The trials focused on evaluating the effectiveness of measurement-based care using electronic patient-reported outcomes and clinical decision support to improve the management of symptoms during and following cancer treatment. We also studied the process of implementing this large new program in diverse settings to inform its adoption and sustainability. A mixed methods approach used EHR data to characterize the sample and evaluate outcomes, while interviews and structured surveys provided information in implementation fidelity and barriers. Delivery of the intervention itself (both ePRO collection and EHR-based clinical decision support) and pragmatic data capture to evaluate outcomes required study teams to fully leverage the capabilities of the EHR and emphasized user-centered design of digital workflows^37^ and an understanding of patient and clinician-engagement and technology uptake.^38^ Implementation science frameworks guided the deployment of strategies to scale up an evidence-based intervention in routine practice settings, and informed the measurement of the implementation outcomes, specifically reach, adoption, and maintenance/sustainment.^39^

Noting the potential strengths of both informatics-based tools and implementation science principles, we assembled a group of IMPACT researchers from CI and IS backgrounds to work together to achieve the projects’ aims. The CI-IS team also included experts in patient-reported outcomes and patient engagement. This multidisciplinary group convened to determine case studies that described distinct, complementary, and synergistic strategies that CI and IS offer to support electronic symptom monitoring and management.

The IMPACT CI-IS team met 16 times throughout 2021 to January 2023 and engaged in open discussions according to a meeting agenda, moderated by the lead author, to achieve the following aims: (1) classify informatics-based tools used in all RCs; (2) identify challenges in developing, deploying, and evaluating each strategy from the perspectives of CI and IS; and (3) critically appraise similarities and differences in CI and IS approaches that hindered or facilitated achievement of the overall goals of the IMPACT Consortium. Meeting notes were compiled and synthesized to address the aims. Finally, the team collaboratively generated lessons learned in service of improving interdisciplinary collaborative research between CI and IS. Results and lessons learned were reviewed and vetted by the IMPACT Publications Committee. The discussions were Institutional Review Board exempt as this was deemed not human subjects research.

## Results

RCs’ aims, study designs, populations, and CI tools used are displayed in Table S1. After describing the IMPACT interventions and the implementation strategies, examples of CI-IS integration are provided for each RC for exemplars of CI-IS integration. CI tools were developed and iteratively refined as implementation proceeded, incorporating patient and clinician feedback to improve their usability and strengthen uptake and sustained use.

### Exemplar 1: the Enhanced, EHR-facilitated Cancer Symptom Control Research Center (E2C2)

E2C2 is an ongoing population-level pragmatic clinical trial that uses a cluster-randomized, stepped wedge design with each step consisting of 3 “clusters,” described below.

#### Background

The E2C2 trial sites include 4 geographic regions (Southwest WI, Northwest WI, Southeast MN, and Southwest MN) as well as eleven specialty practices housed within the Mayo Clinic’s Rochester destination. Each of the regional clusters includes the free-standing and community hospital-based medical oncology clinics within the Mayo Clinic Health System. All sites utilize the Epic EHR, however all had transitioned from 3 different, de-centralized EHRs to the unified, Enterprise-wide Epic EHR.

#### Intervention

Patients were assigned ePROs measuring the 6 SPPADE, domains: sleep disturbance, loss of physical function, pain, anxiety, depression, and energy deficit (fatigue) to remotely monitor patients’ symptoms between visits. These assessments could be completed via the patient portal, tablets in the clinic (if ePRO assignment corresponded to a clinic visit), or via interactive voice response (IVR) telephone calls that allowed patients to input their answers via telephone. Patients who reported symptoms were queried about their receptivity to receiving self-management information via their portal or mailed print materials. Those with the most severe symptoms received a phone call from a research coordinator offering to schedule a time for the patient to discuss their symptoms with a Symptom Care Manager (SCM). Clinicians also had the option to directly refer patients to SCMs via InBasket messaging in Epic or telephone, and SCMs could communicate with clinicians about patients’ goals, barriers, and treatments.

#### Implementation strategies

Clinicians were provided Epic-based means of reviewing patients’ numerical rating scales (NRSs) for the 6 SPPADE domains. Synopsis and flowsheet options were available for viewing symptom scores in the Epic Synopsis view, absent clinician-directed implementation, or engagement efforts to promote use. CI tools included a soft stop alert, and options for personalizing the autofill of SPPADE scores in clinical notes. Clinicians were also offered options for ordering evidence-based treatments to address the SPPADE symptoms via pre-configured order sets.

Clinician champions received brief training to serve as super users for their practice and promote ongoing engagement with the intervention via brief presentations at their tumor group meetings. Practices received monthly newsletters showing the proportion of patients reporting moderate or severe symptoms within each practice over the last month to enable comparison across practices. These newsletters were later revised so that each practice only saw trends in their own practice’s data.

#### Research team challenges and lessons learned

Initially, few patients completed ePROs and used CDS. Practice champions were constrained in promoting CDS use due to competing clinical demands, logistical challenges impeding “at the elbow” support, and lack of EHR fluency despite training. The audit and feedback strategy was felt to be inappropriate by Medical Oncology leadership, as initially conceived, given the potential to exacerbate clinician burnout and frustration with the EHR. Attenuated audit and feedback (eg, sharing reports with practices that show trends in patient symptom severity over time for only their own practice as a whole) has proved to have little effect on use of the E2C2 EHR tools: alerts, autofill clinical notes, and preconfigured order sets.

However, over time, several fruitful approaches that were not initially conceived as CI implementation efforts proved valuable. First, we found leveraging the embedded institutional information technology (IT) training and support infrastructure to be an economical and targeted means of promoting use of some CDS. For example, IT specialists have proved invaluable in customizing clinicians’ notes and orienting them to the E2C2 Synopsis view. Second, our efforts to engage institutional informatics and EHR leadership early in the E2C2 design process had a similarly higher than expected return. Buy-in from these influencers afforded tacit yet impactful endorsement of the project and secured support across levels of EHR governance. Third, E2C2 efforts to proactively and differentially partner with a broad range of stakeholders (eg, nurses and nurse managers, clinic desk operations staff, and palliative care providers) to accommodate their EHR and clinical workflows promoted project acceptance and a level of good will that was vital in later stages of the project.

### Exemplar 2: the Northwestern University IMPACT Research Center (NU IMPACT)

*NU IMPACT* is a type 2 hybrid effectiveness-implementation study using a cluster-randomized stepped-wedge trial design (to test the impact of implementation strategies), with an embedded patient-level randomized trial design (to test effectiveness of the intervention), that takes place at Northwestern Memorial HealthCare Corporation (NMHC), an integrated academic 11-hospital health system affiliated with the Northwestern University Feinberg School of Medicine, spanning areas of central, west, and north metropolitan Chicago.

#### Background

NMHC provides oncology services to over 18 000 unique patients yearly. Outpatients receiving cancer treatment and those in post-treatment survivorship are eligible to participate. NU IMPACT is recruiting an ethnically diverse sample, and about 40% of the Hispanic participants will either be Spanish monolingual or endorse Spanish as a language of preference.

#### Intervention

All participants completed the ePRO assessment in Epic (via the patient portal, associated app, hyperlink, or entered by staff after patients complete a paper form). An invitation to complete the ePRO assessment was sent to patients 72 hours prior to a scheduled oncology visit via the patient portal. The ePRO assessment consisted of Patient-Reported Outcomes Measurement Information System^®^ (PROMIS^®^) measures (ie, Depression, Anxiety, Fatigue, Pain Interference and Physical Function), along with a checklist in which patients endorse supportive care needs for which they want to be contacted by a healthcare professional (social worker or dietitian). The ePRO assessment takes approximately 6-7 minutes to complete. Severe scores and endorsed care needs triggered clinical alerts sent via an Epic InBasket to patients’ oncology clinicians as part of usual care.

#### Implementation strategies

NU IMPACT investigators collaborated closely with NMHC Epic administrative and clinical teams to design, iteratively refine, and implement the ePRO system. Implementation strategies were numerous and included integration into clinical workflows, engaging with system and clinic leadership, identifying, and leveraging champions, training, and educating clinicians, providing severe symptom alerts within Epic, and conducting audit and feedback to strengthen inclusion of PRO data in patient–clinician shared decision-making.

#### Research team lessons learned

Barriers to widespread adoption of ePRO system included that health system patients were not registered for the patient portal. Relatedly, while NU IMPACT patient-facing materials and the PROs were available in Spanish, the NMHC patient portal was only available in English, which created challenges for Spanish-speaking patients. Also, unregistered patients did not automatically receive their assessments, and in-clinic assessment options were limited during the COVID-19 pandemic. To avoid introducing bias, several system-wide strategies were developed to improve uptake of the patient portal and promote in-clinic completion as a backup. Most notably, responding to staff feedback, NU IMPACT shortened the ePRO assessment mid-implementation (switching from PROMIS computer adaptive tests to PROMIS short form measures). This change allowed for (1) more in-clinic assessment options, including during the COVID-19 pandemic (eg, clinics who preferred to administer the assessment on wipeable laminated cards to patients who had not completed it electronically in advance) and (2) administration via the local Epic patient portal application (vs just website access).

### Exemplar 3: the Symptom Management IMplementation of Patient Reported Outcomes in Oncology (SIMPRO) Research Center

SIMPRO deployed patient-reported outcomes assessment and symptom management intervention in 6 cancer centers in the northeastern and southern United States. Sites were selected because they treated a diverse patient population based on age, race/ethnicity, and rurality, and because they all use the Epic EHR system.

#### Background

SIMPRO partnered with Epic to develop and deploy eSyM, an ePRO-based, EHR-integrated symptom management tool based in part on National Cancer Institute’s Patient-Reported Outcomes version of the Common Terminology Criteria for Adverse Events (PRO-CTCAE^®^). eSyM enables patients and clinicians to track and react to symptomatic adverse events following cancer-related surgery or initiation of treatment for gastrointestinal, thoracic, or gynecologic cancers.^29^

#### Intervention

Participants receive (1) automated symptom questionnaires via the Epic-linked patient portal at pre-specified intervals, (2) alerts to contact their care team for severe symptoms, and (3) access to self-management tip sheets. The care team monitors the patient cohort via 2 dashboards and 4 workbench reports that help team members track and manage symptom reports. Deployment of eSyM required careful alignment with informatics staff from the Beacon oncology order entry, Healthy Planet patient registry, and MyChart patient portal teams.

#### Implementation strategies

Prior to eSyM deployment, processes for monitoring symptoms via ePROs were nonexistent at 5 centers and in the early phase of implementation at the sixth center (Dana-Farber Cancer Institute). The Patient Reported Data (PRD) program at Dana-Farber was collecting symptomatic adverse events across 15 PRO-CTCAE domains within the EHR during medical oncology clinic encounters. Additionally, the PRD program conducted a feasibility study of symptomatic adverse event reporting between clinic encounters among patients prescribed oral chemotherapy.

#### Research team challenges and lessons learned

Success required careful communication and coordination of CI strategies. While eSyM content was standardized, differing instances of Epic across the 6 health systems meant that engagement from the health system’s CI build teams and guidance from Epic Systems was needed to adjust the deployment for each setting. Staggered deployments allowed the implementation team to work closely with each health system’s CI group. Customized configuration and training were required to address the challenges of provider and patient engagement given the variety of implementation sites and cultures involved.

Early implementation lessons gained from this experience, and from validation studies conducted during the development of PRO-CTCAE served as the basis for eSyM deployment. Three key improvements made for eSyM included: (1) expanding collection of symptomatic adverse events to include surgical and medical oncology patients, (2) gathering symptom data between clinic encounters, and (3) creating tools that help track patient populations over time and foster proactive responses to severe symptom reports.

Ongoing challenges include the need to plan for sustainability as Epic instances are upgraded and the need to improve patient and provider engagement in each health system. Further study is underway to understand factors predicting patient response to eSyM questionnaires and provider use of eSyM reports. However, the lessons learned by deploying CI tools within an IS study underlie the program’s success to date. Specifically, investigators familiar with both CI and IS strategies met regularly across all 6 sites to develop and refine the CI approach. Epic was involved early in the process to define the capabilities and functions of the EHR and to determine which ones could be modified to meet the needs of eSyM implementation. We look forward to completing enrollment for the study and learning more about how to sustainably deploy symptom management in the ambulatory oncology practice. The CI tools used in eSyM are being employed to support sustainment.

## Discussion

The IMPACT exemplars in each RC illustrate mutually beneficial and overlapping CI tools and IS methods. The use of CI tools as implementation strategies to support adoption and sustainment of systematic symptom assessment via ePROs in ambulatory oncology and an associated symptom management intervention underscores the conceptual and practical connection between CI and IS. Although diverse in context (academic vs community), setting (single site vs multisite), site readiness (established vs nascent ePRO teams), and integration (full vs partial EHR integration), all shared a similar objective—optimizing symptom management for individuals diagnosed with cancer by leveraging the EHR platform.

There are several main takeaways from CI and IS integration illustrated by the IMPACT examples. First, key stakeholders must be identified early in the process from both CI and IS, including leaders in operations, clinicians, and patient advocates. In addition, implementation teams must identify the routes of implementation, such as the EHR-based deployment approach being used by each RC. The EHR can be beneficial as a platform for systematic symptom assessment and management. However, not all health systems have access to an EHR, or use hybrid EHR/paper systems. Other CI tools have similar challenges related to system-wide deployment. Once a CI implementation effort is initiated, the team should review progress against expected process and outcomes metrics and refine the implementation strategy as needed.

Second, once implementation teams are assembled, they must determine their strategic frameworks—whether borrowing from CI, IS, or both—that will be used including timeline, metrics of successful implementation, and focus of patient, provider, and healthcare system levels of engagement. Our IMPACT RC examples highlight the relevance of internal factors and the importance of establishing institutional readiness for electronic symptom management intervention implementation.

Third, the pain points felt by CI and IS researchers remain under-recognized (eg, competing prioritization of timelines, securing staff availability) resulting in gaps in uptake and adoption. Developing a shared culture in which CI and IS researchers use a common vocabulary around implementation could foster more effective discussions, leading to smoother implementation and improved outcomes. Further research that incorporates principles and strategies from community engagement can inform how IS and CI stakeholders can be intentional about aligning strategies to meet each other’s objectives.^40^

Lastly, IMPACT’s strategically applied investments (time, leadership, and personal capital) informed by IS have catalyzed IS-forward approaches to CI interventions. NCI funding for IMPACT has been a facilitator in the development and evaluation of CI implementation strategies as well as sharing of best practices and pitfalls along the way. These include foreshadowing challenges with site or provider engagement, limitations of the EHRs, or visual representation of ePRO reports. Moreover, systematically mapping determinants to CI implementations using a framework such as CFIR, a common practice in IS, enables research teams to ensure that all stakeholder “voices” are heard, by identifying potential unforeseen gaps. This in-turn leads to the development of implementation strategies to address identified barriers, which influence behavior change at the system and individual levels.

We report the following limitations. We were able to leverage a moderate-to-strong degree of site readiness within each research center. Starting implementation at sites with a general awareness of ePROs and their value proposition greatly facilitated engagement of single and multi-site healthcare systems in this effort. For smaller institutions that may not have access to CI and/or IS expertise, similar implementation may not be feasible. In those settings, we advise pragmatic standards for ePRO implementation in ambulatory oncology such as language concordance with the target population, multimodality access points for patient response, and a carefully outlined plan for timely intervention for moderate or severe symptomatic adverse events. Table S2 displays a consolidated set of recommendations grouped by ecological level.

Given this was a case study report, the findings are transferable^41^ more than they are generalizable and are therefore intended to describe experiences and insights that readers may apply to their particular circumstances. Second, we may not have exhaustively captured the experiences and insights derived from each center’s implementation; yet we triangulated at multiple levels to ensure we comprehensively and accurately captured the findings and points of discussion. Recognizing the significance of grant funding in facilitating IMPACT Consortium studies, we must consider issues of sustainability in the real world. Although each Research Center provided implementation support made possible by the grant funding, a significant amount of support came from the participating healthcare systems, reflecting buy-in with the implementation effort. The SIMPRO eSyM tool has been adopted by Epic, a mainstay in EHRs, and further investigation will determine the extent of adoption and adaptation to local context/resources needed to sustain this effort. Lastly, we acknowledge the act of implementation may alter perception of patient-reported outcomes in clinical practice favorably or unfavorably. Fortunately, qualitative data obtained at baseline for each IMPACT consortium study provide robust benchmarks for comparison of change in perspective/behaviors because of each implementation effort.

## Conclusion

CI and IS each focus on the effectiveness that socio-technological tools have on care delivery and outcomes. CI approaches and their evaluative frameworks are focused on technical aspects and standardization to improve care quality and efficiency overall whereas IS is grounded in hypothesis-driven study designs, viewing CI as one of many strategies needed to improve EBI implementation, while also considering additional strategies and potential adaptations needed to ensure sustainment. Despite their contrasts, CI and IS mutually shape each other, and efforts from the IMPACT Consortium revealed that CI and IS methodologies and perspectives have relied on each other and that domain experts have had to translate their worldviews to effectively collaborate. We believe these 2 fields have many future opportunities to contribute to the success of EBI deployment and sustainability. Within learning health systems as such, we call for ongoing dialogue and expansion of IS theories, models, and frameworks to better incorporate CI that promotes additional shared learning as well as identifying gaps between CI and IS and relevant solutions for bridging these robust research communities.

## Supplementary Material

ooae081_Supplementary_Data

## Data Availability

Descriptive and process information is provided in this manuscript; no quantitative or qualitative data are presented.

## IMPACT consortium group members

David Cella, PhD; Andrea Cheville, MD, MSCE; Michael J. Hassett, MD, MPH; Raymond U. Osarogiagbon, MBBS, FACP, MD; Deborah Schrag, MD, MPH; Sandra L. Wong, MD; Barbara L. Kroner, PhD, MPH; Ashley Wilder Smith, PhD, MPH; Lisa DiMartino, PhD; Sofia Garcia, PhD; Joan Griffin, PhD; Roxanne Jensen, PhD; Sandra Mitchell, PhD, CRNP; Kathryn Ruddy, MD, MPH; Justin D. Smith, PhD; Betina Yanez, PhD; Jessica J. Bian, MD; Don S. Dizon, MD, FACP; Hannah W. Hazard-Jenkins, MD, FACS; Mary-Anne Ardini; Paige Ahrens, MS; Jessica Austin, PhD; Fiona Barrett; Michael Bass, MS; Megan Begnoche, RN, MSN; September Cahue, MPH; Kimberly Caron, RN, BSN, CCRC; Linda Chlan, PhD, RN; Ava Coughlin, MAEd; Christine Cronin; Samira Dias, MPH; Nicolas Faris, MDiv; Anne Marie Flores, PhD, PT; Martha Garcia; Karla Hemming, PhD; Jeph Herrin, PhD, MS; Christine Hodgdon, MS; Sheetal Kircher, MD; Kurt Kroenke, MD, MAC; Veronica Lam; Nicola Lancki, MPH; Quan H. Mai, MS; Jennifer Mallow, PhD, FNP-BC; Nadine J. McCleary, MD, MPH; Wynne Norton, PhD; Mary O'Connor, MS; Deirdre Pachman, MD; Loretta Pearson, MPhil, CCRC; Frank Penedo, PhD; Jewel Podratz, MBA; Jennifer Popovic, DVM, MA; Liliana Preiss, MSE; Parvez Rahman, MHI; Sarah Minteer, PhD, MA; James Reich, PMP; Joshua Richardson, PhD; Kimberly Richardson, MA; Jennifer Ridgeway, PhD; Lila Rutten, PhD; Karen Schaepe, PhD; Denise Scholtens, PhD; Tiana Poirier-Shelton, MPH; Philip Silberman, MA; Jaclyn Simpson, MBA; Laura Tasker, BS, RT(N); Nathan Tesch, MS; Cindy Tofthagen, PhD; Angela Tramontano, MPH; Benjamin D. Tyndall, PhD; Hajime Uno, PhD; Firas Wehbe, MD, PhD; Bryan Weiner, PhD, MA.
