# Supplementary material for: Bridging clinical informatics and implementation science to improve cancer symptom management in ambulatory oncology practices: experiences from the IMPACT consortium
Source: JAMIA Open. 2024 Sep 4;7(3):ooae081. doi: 10.1093/jamiaopen/ooae081 (PMC11373565; doi:10.1093/jamiaopen/ooae081)
Supplement: ooae081_Supplementary_Data [file ooae081_supplementary_data.zip › 2023-05-10_supplementaryTable01.docx]

**Supplementary Table 1: Overview of the Research Centers**

| Research Center | Study Design | Aims | Target Population/Enrollment | CI Tools |
| --- | --- | --- | --- | --- |
| Enhanced, EHR-facilitated Cancer Symptom Control (E2C2) | Cluster randomized stepped wedge trial | - Reduce SPPADE symptom (sleep disturbance, pain, loss of physical function, anxiety, depression, and energy deficit) scores - Improve physical function and symptoms, enhance quality of life (QoL), increase adherence to cancer therapies and reduce unplanned hospitalizations and emergency department visits | All patients being seen for a solid or liquid cancer at a Midwest Mayo Clinic Health Systems (MCHS) site or for a solid tumor at the Mayo Clinic Rochester tertiary practice irrespective of phase of disease. | - EHR (Epic) - EHR-based CDS - ePROs - Patient portal - Alerts/reminders - Dashboards |
| Northwestern University (NU) IMPACT | Cluster randomized modified stepped wedge Type 2 hybrid effectiveness-implementation study | - Implement a fully EHR-integrated oncology symptom assessment and management program - Evaluate the impact of the program; Identify facilitators and barriers to implementation and disseminate to other health systems | Adult oncology outpatients (in treatment with curative or non-curative intent, , or in post-treatment survivorship) | - EHR (Epic) - Patient portal - Alerts/reminders - Web-based symptom self-management intervention - ePROs |
| SIMPRO | cluster randomized stepped-wedge Type 2 hybrid effectiveness-implementation study | - Adapt existing ePRO symptom management systems and integrate them into the EHR - Determine the effectiveness of an EHR-integrated ePRO symptom management system on health outcomes; - Evaluate the facilitators and barriers to implementation of an EHR-integrated ePRO symptom management system from the patient, clinician, and organizational perspectives | Surgical and medical oncology patients | - EHR (Epic)-integrated symptom management tool - eSyM app that is ePRO-based |
