# Supplementary material for: Bridging clinical informatics and implementation science to improve cancer symptom management in ambulatory oncology practices: experiences from the IMPACT consortium
Source: JAMIA Open. 2024 Sep 4;7(3):ooae081. doi: 10.1093/jamiaopen/ooae081 (PMC11373565; doi:10.1093/jamiaopen/ooae081)
Supplement: ooae081_Supplementary_Data [file ooae081_supplementary_data.zip › 2023-12-06_supplementaryTable02_CLEAN.docx]

**Supplementary Table 2: Multi-level Implementation Considerations for EHR-based ePRO Systems for Routine Cancer Symptom Monitoring and Management[33-39]**

| Level | Implementation Consideration |
| --- | --- |
| Patients | - Extent to which portal is integrated into patient-provider interactions to enhance bidirectional communication and information exchange (e.g., patient/provider secure messaging is available within the EHR, ability for patient/provider to view ePRO trends) - Patient expectations/norms that electronic symptom reporting is being acted upon as a component of visits (e.g., ePRO reports are routinely reviewed during patient encounter; self-management instructions are integrated into visits) - Expectation that questionnaires may be delivered using different administration approaches that offer flexibility in responding to survey (e.g., self-report via tablet, portal, IVR, or interviewer administered in-clinic) - Positive feedback for providing symptom reports to incentivize ongoing reporting (e.g., shared patient-clinician review of ePRO values/trends during appointments; acknowledgement of reporting by clinical staff; dashboard integrating ePRO and treatment data) - Tools, measures, and other materials are offered in formats that are flexible and responsive to the needs of patient subgroups who speak languages other than English, have lower digital literacy or barriers to broadband internet access (e.g., self-management materials available in a wide range of languages, materials are easy to access through widely compatible software such as PDFs, portal navigation prompts are intuitive and available in patient’s preferred language) - Availability of customer technical support for portal access, password reset or other troubleshooting (e.g., on-premise help desk, evening/weekend technical support, one-time password reset tokens) - Portal access and symptom surveillance measures are compatible with a wide range of devices and response options (e.g., bring your own device [BYOD], QR code, interoperability with legacy devices/systems) |
| Providers (Physicians, Advanced Practice Providers, Registered Nurses and Interdisciplinary providers [e.g. rehabilitation, nutrition, pharmacy, social work]) | - Consistency and patterns of interfacing with EHR to enter, review, synthesize ePRO reports with other EHR components, and utilize ePRO data in clinical decision-making (e.g., interpreting trends in ePRO scores in the context of recent pain medication adjustments) - Clinician attitudes and expectations towards portal usability and EHR functionality (e.g., arduous and frustrating vs. potential to be helpful and efficient in supporting care delivery) - Clinicians are proficient and flexible in the use of EHR functionalities and view filtering options that support the clinical workflows and role functions (e.g., dot phrase functionality, flowsheets, interactive graphical displays, accordion views) - Discipline/role-specific workflows are established within the care team for reviewing and following up on ePRO data, including alerts - ePROs-linked decision-support, symptom management guidelines, and databases of referral resources are integrated into care pathways and the EHR |
| Institution(s)/Health Systems(s)/Practice Site(s) | - Ability to customize EHR interface to clinician and patient needs, specifications, workflows, and preferences at the institution-, department-, and individual-level (e.g., guidelines, algorithms, order templates, alerts, and other decision support are tailored to practice site/team preferences) - Governance, oversight, and resources to support activities related to proposing, building, assigning, and maintaining ePROMs, in and across EHR systems - Logistics and staffing for EHR education and support include both centralized and decentralized services that facilitate ePRO-based symptom screening and management (e.g., clinically embedded EHR specialists) - Availability of symptom management resources both electronically and at the point-of-care (e.g., patient education; referral resources and pathways) - Organizational culture, norms, and clinical performance standards prioritize ePRO collection, including clear performance expectations that providers act on ePRO data as a standard of care and standard operating procedures regarding the timing and type of provider response to actionable ePRO values - Institutional and department-level coordination and alignment of ePROs to reduce or address potential patient and clinician burden (e.g., selection of domains, standardized measures, administration cadence [both at visits and between visits] interpretation thresholds and alerts are coordinated across departments to eliminate or reduce overlap) |
| Health Policy | - Models for reimbursement/financial offset for symptom surveillance (e.g., through bundled payments, case rates, and payer incentives, new CPT codes for remote therapeutic monitoring and telehealth to reduce ER visits and adverse outcomes) - Availability of quality standards (including endorsement of specific PROMs) from professional groups to support ePRO-based symptom screening and management (e.g., ASCO, ONS, APOS, NQF) - Availability of accreditation standards/requirements that mandate symptom screening and management (e.g., ACOSOG, Commission on Cancer, The Joint Commission, Magnet) |

*Note: ASCO = American Society of Clinical Oncology, ONS = Oncology Nursing Society, APOS = American Psychosocial Oncology Society, NQF = National Quality Forum, ACOSOG = American College of Surgeons Oncology Group*
